# [Retracted] Downregulation of microRNA-206 suppresses clear cell renal carcinoma proliferation and invasion by targeting vascular endothelial growth factor A

**DOI:** 10.3892/or.2024.8717

**Published:** 2024-02-26

**Authors:** Yi Cai, Hanzhong Li, Yushi Zhang

Oncol Rep 35: 1778–1786, 2016; DOI: 10.3892/or.2015.4538

Following the publication of the above paper, it was drawn to the Editor's attention by a concerned reader that there appeared to be several instances of overlapping data panels comparing between the Transwell invasion and migration assay images shown in Figs. 2E and 4G, such that data which were intended to show the results from differently performed experiments were apparently derived from a (much) smaller number of original sources.

Given the number of cases of overlapping data panels both within and between this pair of figures in the article itself, the Editor of *Oncology Reports* has decided that this paper should be retracted from the Journal on the basis of a lack of confidence in the presented data. The authors were asked for an explanation to account for these concerns, but the Editorial Office did not receive a reply. The Editor apologizes to the readership for any inconvenience caused.

